# Nature Studies.—IV

**Published:** 1910-02-12

**Authors:** 


### The Practitioner's Relaxations.

### NATURE STUDIES.—IV.

By a MAJOR, R.A.M.C.

Winter on the Downs—Field Fares—Rustic Piety—First Leaves to Fall—The Chestnut—White Rabbits—Starlings in India—Minak—Going South.

Here on the downs winter is upon us. The northerly winds make it very cold. There is frost every night, and, though the sun is often bright, the frost scarcely leaves the high ground all day. Often clouds come up as if there was to be snow. The field-fares have come in in good numbers. For weeks past I have seen a few in every direction feeding on the great straggling fences that shelter the fields beneath the downs and reach far up the downs as boundary hedges. On the eleventh or twelfth of November I noticed a perceptible increase of their numbers; I saw them in flocks with song thrushes and missel thrushes and, I think, redwings. But, as the flocks were high in some long strips of fir plantations that run along the downs, and I was riding, I could not for certain identify the redwings. A few days later I saw redwings in the great penning behind my house.

The field-fares feed chiefly in the hedgerows. They eat the fruit of the wild rose. This is, I think, their favourite hedgerow fruit. They also peck about in the grass fields and in water meadows.

### The Berry Crop.

It is said that a big berry crop portends a hard winter. Rustic piety holds that the Almighty, intending to visit us with severe weather, provides in advance a supply beyond the ordinary for His birds. If there be this connection between berry crop and winter weather the signs are for a hard time this year, for the crop is abundant.

Hard frost, the second this winter, ushered in the King's birthday. The thermometer marked 18 degrees below freezing point, and much leaf fell. The ash is the first tree to lose leaf on the coming of winter; with the first severe frost down it comes, and at the same time much of the leaf of the horse chestnut. The latter lose leaf less completely. Every leaf may fall from one chestnut tree, and yet on another near by the main of the leaves may remain—though brown, dry and withered, hanging to the branches.

On the day of the first hard frost, it was in the latter part of October, I noticed that in the paddock on the south side of our churchyard a certain chestnut tree had been stripped bare. The leaves lay thick upon the ground. They had dropped straight down, and lay like a thick carpet round the trunk, for the air was too still to drift them. In the same field are other trees of the kind, and they, not more sheltered, had kept most of their foliage. A few days later, in a garden some miles away, a friend of mine pointed out to me that of a row of six magnificent chestnuts one was absolutely leafless, though another still had its leaf almost intact. In neither instance was there obvious reason. Perhaps it may be a difference of constitution; using that word in the same loose way that it is often used of human beings. The frost of the ninth stripped the poplars. In the early morning I was walking by a river in a long meadow that grows a row of great poplars along the river bank. The leaves were falling heavily and thickly like snow flakes on a still day.

### Albino Rabbits.

I was shooting that day in a covert some eighteen miles from my house. Amongst the bag was a rabbit with a deal of white marking. We saw another rabbit almost white. I remember that, when I was a boy, this cover was remarkable for holding variegated rabbits. The explanation was that the owner, who had, I suppose, a fancy for a varied bag, had had a number of tame rabbits turned out. So any day black, white, sandy, and variegated rabbits might be seen running across the rides or feeding on the turfy spaces. It is a good many years since that time, and now the rabbits have practically all reverted to the common wild type. But still it seems an odd one or two carries back through many generations to the tame strain. The rabbit we shot showed no sign, except in colour, of departure from the wild type; no extra size nor lopping of ear.

### Bird Life on the Downs.

The greater cold has sent the big flocks of starlings from the downs. I see only smaller flocks and scattered parties of half a dozen or a dozen birds. Those, perhaps, are the locally bred birds; the larger flocks migrating birds. Perhaps the larger flocks would now be found in the valleys or the meadows by the chalk streams; but the nearest stream is some miles away, and I have not been down into the valley lately. No doubt many have gone on across the seas. In the plains of India during the cold weather there are great flocks of starlings. I have often seen them at sunset flying across the Ganges Kadar, and the marshes of the United Provinces. Starlings do not breed in the plains of India, I believe, and certainly not in any numbers. Their place is taken by the minak and the rose-winged starling—I think I have the name right—a handsome bird, much like the starling in habits. Both kinds follow the herds of cattle to feed on the insects they disturb and the parasites that drop from them; just as our starlings follow the flocks of sheep on the downs.

The minak is very fond of a certain small kind of grasshopper. I had a tame one once that would follow me about my compound and dash at, catch, and eat the grasshoppers I disturbed in walking through the grass.

The lines of emigration of birds from England do not lead to India. The starlings seen there are, I presume, bred in the temperate latitudes of Asia. Our flocks would go on to south of the Mediterranean.

Larks have decreased much in the last few days on the downs and on the higher fields. I still see a good many in the lower fields, but none sing now. No doubt a good many of them have gone south, though plenty stay with us all the year. If there comes hard frost and snow the larks draw down in great numbers to the water meadows, and there find food on spaces kept free of snow by the overflow of water from the ditches. I remember well that the first shot I ever fired was at a flock of larks feeding on a wet place amongst the snow in the meadows. I was thirteen years old, and used an old single-barrelled muzzle-loader and killed fourteen larks at one discharge. It was a very severe winter.

